# Long‐Term Memory Engrams From Development to Adulthood

**DOI:** 10.1002/hipo.70032

**Published:** 2025-08-06

**Authors:** Abigail L. Yu, Laura A. DeNardo

**Affiliations:** ^1^ Department of Physiology University of California Los Angeles Los Angeles California USA; ^2^ Neuroscience Interdepartmental Program University of California Los Angeles Los Angeles California USA; ^3^ Department of Neurobiology University of California Los Angeles Los Angeles California USA

**Keywords:** development, infantile amnesia, memory consolidation, memory engram, synaptic plasticity

## Abstract

Memories formed in adulthood can last a lifetime, whereas those formed early in life are rapidly forgotten through a process known as infantile amnesia. In recent years, tremendous progress has been made in understanding the memory engram—the physical trace of a memory in the brain—and how it transforms as memories evolve from recent to remote. This review focuses on engram cells and examines their roles in memory encoding, consolidation, retrieval, and forgetting from development to adulthood. We concentrate on four key brain regions: the hippocampus, the retrosplenial cortex, the medial prefrontal cortex, and the anterior thalamic nuclei. We first focus on the adult brain, highlighting recent studies that reveal the distinct contributions of engram cells in each brain region, with particular emphasis on synaptic plasticity and memory consolidation. We then explore how coordinated activity across these regions supports long‐term memory. In the second section, we review emerging knowledge of engram cells in the developing brain, examining how developmental differences in their functions contribute to infant memory generalization and infantile amnesia. Compared to adults, much less is known about how, or to what extent, early‐life memories undergo consolidation. In the final section, we synthesize current knowledge of memory consolidation and retrieval in the adult brain with what is known about the development of the four brain regions we discuss. We then propose key directions for future research. In sum, this review brings together recent findings that deepen our understanding of the dynamic changes in memory engrams that underlie consolidation and long‐term storage and explores how developmental differences may shape the maturation of memory processes.

## 1 Introduction

We rely on memories of past experiences to make decisions and navigate dynamic environments. While some memories can last a lifetime, those formed very early in life are short‐lived, a phenomenon referred to as infantile amnesia (IA). The mechanisms through which the brain stores enduring memories in adults, and how these processes change across development, have been a major focus of study, especially in recent years as new tools have accelerated progress (Josselyn and Tonegawa 2020; Tonegawa et al. 2015, 2018). In this review, we discuss recent studies that have revealed novel insights into how the brain stores long‐term memories. By aligning studies of the developing and the developed brain, we extend new hypotheses about the mechanisms underlying IA and highlight key directions for future study.

As we have experiences, our brain binds together sensory information into a representation of a discrete event that occurred in a specific place and time. Then, our brain consolidates that representation into a network that is optimized to promote retrieval when an individual encounters meaningful reminders (Preston and Eichenbaum 2013). Research dating back to the late 19th century revealed that the brain's method of storing memories changes over time. The experimental psychologist Théodule Ribot observed that patients who had suffered trauma often lost memories of events leading up to the trauma, including the years immediately preceding it, while older memories remained intact. Based on these observations, he formulated Ribot's Law, which describes the process of retrograde amnesia and suggests that newer memories are more susceptible to disruption than older ones (Ribot 1882). In subsequent decades, case studies of patients who underwent experimental brain surgeries, including Henry Molaison (Patient H.M.), provided additional insights into the brain mechanisms driving these time‐dependent changes (Scoville and Milner 1957). The experiences of these patients demonstrated that the hippocampus (HPC) and neighboring structures within the medial temporal lobe are critical for the initial formation and storage of episodic memories. Then over time, memories are reorganized into a distributed network of brain regions, particularly within the neocortex. This process, known as systems consolidation, is believed to create a more stable representation of a memory that is better suited for long‐term storage (Tonegawa et al. 2018; Frankland and Bontempi 2005, 2006). Understanding the detailed circuit mechanisms underlying this transition has been a major focus of study in recent years.

The memory engram—the physical representation of a memory in the brain—has been a topic of great interest in memory research. While the nature of the engram was long elusive, the advancement of viral‐genetic approaches in the last 20 years enabled massive strides in our understanding of the engram. Viral and genetic tools that leverage immediate early gene (IEG) expression to target and manipulate cells that are active during an experience enabled the discovery of engram neurons (DeNardo and Luo 2017; Luo et al. 2018). Engram cells are hypothesized to mediate memory encoding, consolidation, and retrieval and are typically characterized by two or more of the following properties: (1) active during learning, (2) undergo enduring physical changes as a result of learning, (3) reactivated during memory retrieval, and (4) required or sufficient to mediate memory retrieval (Tonegawa et al. 2015).

While the functions of engram cells in memory encoding and retrieval have been extensively reviewed, here we focus on recent studies of their roles in memory consolidation. Additionally, we examine new research that has identified novel mechanisms of IA. We focus on four key brain regions: HPC, retrosplenial cortex (RSC), anterior thalamic nuclei (ATN), and medial prefrontal cortex (mPFC). This densely interconnected network has established roles in memory encoding and consolidation in adults (Frankland and Bontempi 2006; Toader et al. 2023; Vann et al. 2009; Maviel et al. 2004; Frankland et al. 2004; Yadav et al. 2024; Trask, Ferrara, et al. 2021), but its functions are only beginning to be examined in development. Through integration of these findings, we describe how they advance our understanding of the basic processes underlying long‐term memory in adults, how memory processes differ during development, and promising new avenues for investigation.

## 2 Mechanisms of Long Term Memory in Adulthood

Forming a stable memory representation—the memory engram—requires experience‐dependent changes to the brain that occur in stages during memory encoding and consolidation (Guskjolen and Cembrowski 2023). Memory consolidation relies on interactions among several interconnected brain regions including the HPC, RSC, mPFC, and the ATN, which are the focus of this review. In this section, we examine the role of each region in long‐term memory in the adult brain and explore how their interactions support memory processes. We begin by outlining the basic anatomy and connectivity of each region, followed by a discussion of the functional mechanisms involved in memory, with a particular focus on engram cells.

### 2.1 Hippocampus

Memories are initially encoded in the well‐characterized trisynaptic circuit of the HPC, which includes three major HPC subregions: the dentate gyrus (DG) and the cornu ammonis (CA) fields CA3 and CA1. The entorhinal cortex (EC), which integrates highly processed sensory and spatial information, projects to DG via the perforant path. Granule cells in DG, located in the superior and inferior blades, project to CA3 through the robust mossy fiber pathway. CA3 pyramidal cells then project to CA1 via the Schaffer collateral pathway. Both CA3 and CA1 pyramidal cells also receive direct input from the EC through the temporoammonic pathway, which forms synapses on the distal dendrites of these cells. The subiculum serves as the primary output of the HPC, projecting back to the neocortex, including to the RSC and PFC. It also projects to the ATN, both directly via the fornix and indirectly via the mammillary bodies (Aggleton et al. 2005).

**FIGURE 1:**
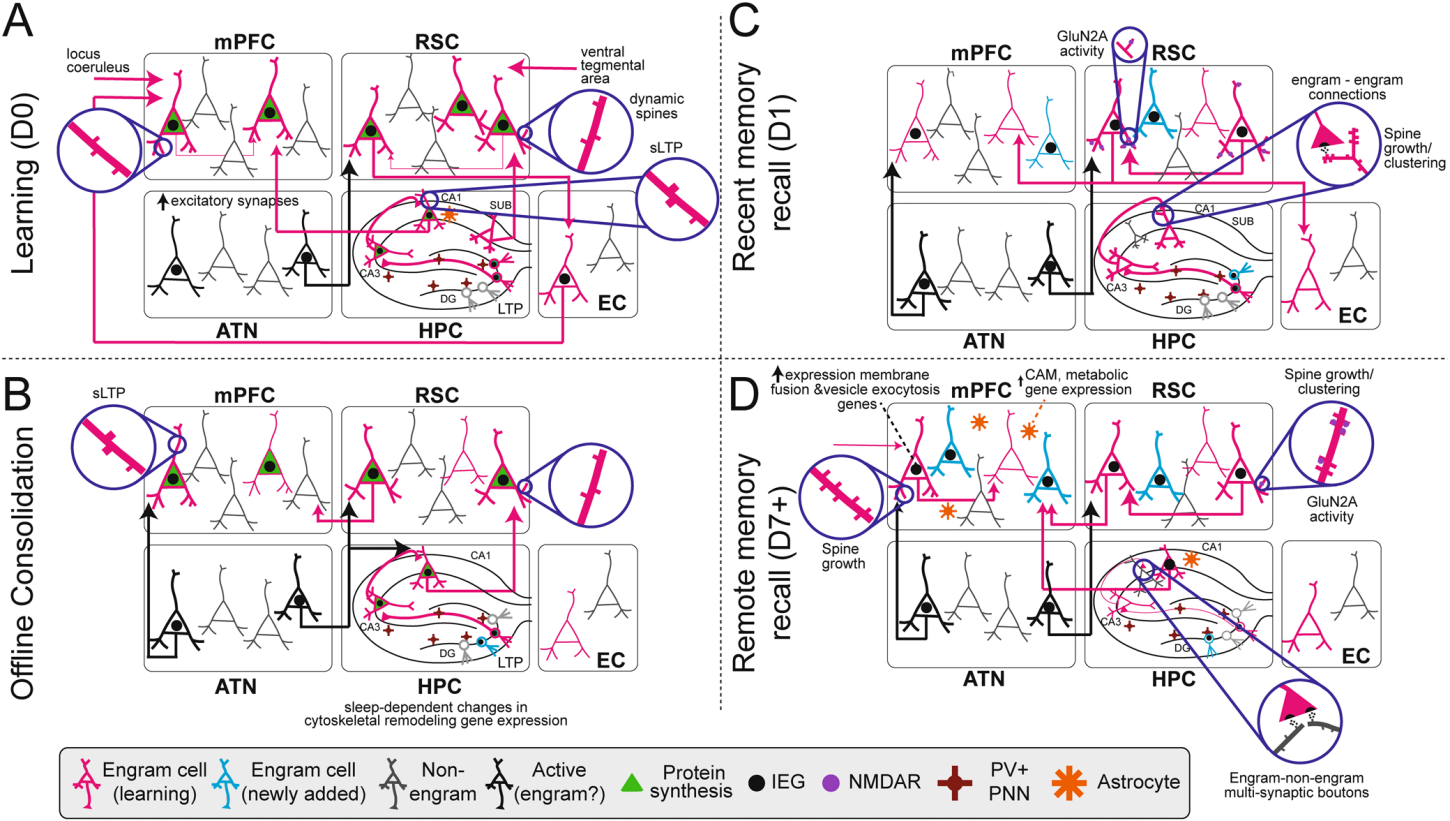
The roles of engram cells in memory encoding, consolidation, and retrieval in adulthood. Summary of recent findings about engram cells in the mPFC, RSC, ATN, and HPC. (A) Processes occurring during learning on day (D) 0. EC‐ and LC‐inputs to mPFC are critical for tagging engram cells. Dopaminergic (Ventral Tegmental Area), HPC, and ATN inputs to RSC are critical for learning. ATN exhibits increased activity, IEG expression, and excitatory synapse density. Structural long‐term potentiation (sLTP) is evident in the hippocampus. Activity in the subiculum‐RSC pathway and astrocyte‐mediated recruitment of mPFC‐projecting CA1 engram cells are important for later memory retrieval. (B) Memory consolidation in the hours following learning, including during sleep, involves sLTP in mPFC and coordinated activity between ATN and mPFC, ATN‐RSC and ATN‐HPC. Additionally, there is incorporation of new neurons into the HPC engram and sleep‐dependent changes in gene expression in HPC engram cells. (C) Recent memory retrieval on D1 after learning involves ATN‐mPFC, ATN‐RSC‐EC, and HPC‐RSC pathway activity, NMDAR activity in RSC, addition of new/clustered dendritic spines in hippocampal neurons, and preferential connections between HPC engram cells. (D) Remote memory retrieval occurring 7 days or more after learning involves the maturation of mPFC engram cells and recruitment of new mPFC neurons to the memory engram, changes in gene expression in mPFC engram cells and mPFC astrocytes, spine growth and clustering in RSC neurons, ATN‐mPFC and ATN‐RSC pathway activity, and enhanced engram‐non‐engram cell synaptic connectivity in the HPC.

Over the last two decades, studies have uncovered the mechanisms that recruit neurons to a memory engram and the roles of HPC engram cells in memory encoding, recall, and specificity. A neuron's intrinsic excitability positively influences its likelihood of being incorporated into an engram (Mocle et al. 2024). The cAMP Response Element‐Binding protein (CREB) plays a key role in this process by regulating neuronal excitability (Park et al. 2020). During learning, HPC engram cells show more coordinated and repetitive activity than non‐engram cells (Ghandour et al. 2019) (Figure 1A). After learning, HPC engram cell activity is both necessary and sufficient to retrieve recently formed memories (Han et al. 2022; Liu et al. 2012; Ramirez et al. 2013) (Figure 1C). Moreover, functionally distinct engrams, genetically defined by the expression of different IEGs, regulate memory specificity. Fos‐expressing ensembles regulate memory generalization through mechanisms that depend on long‐range input from the EC (Figure 1C), whereas Npas4‐expressing ensembles enhance memory specificity by recruiting local inhibition via cholecystokinin (CCK)+ interneurons (Sun et al. 2020). These studies have been instrumental in revealing how neurons are allocated to a memory engram and in identifying the behavioral functions of HPC engram cells during recent memory retrieval. While these studies highlight the importance of neuronal excitability in engram allocation, they do not rule out the critical role of synaptic plasticity in memory consolidation.

Popular theories of long‐term memory emphasize Hebbian plasticity mechanisms. These theories propose that synaptic strengthening between neurons active during memory encoding stabilizes a network of learning‐activated cells, facilitating future memory retrieval (Morris 1999). The maintenance of long‐term changes in synaptic strength—such as long‐term potentiation (LTP) and long‐term depression (LTD)—requires new protein synthesis (Sutton and Schuman 2006). Systemic administration of the protein synthesis inhibitor anisomycin after contextual fear conditioning (CFC) induced retrograde amnesia and blocked learning‐induced synaptic plasticity in DG engram cells (Ryan et al. 2015) (Figure 1A,B). Surprisingly, however, optogenetic reactivation of these cells still triggered conditioned freezing behavior, suggesting that protein synthesis‐dependent synaptic plasticity is not required for memory storage, but rather for successful retrieval of a stored memory (Ryan et al. 2015). Anisomycin also disrupted context‐dependent HPC place field remapping and coordinated cellular activity that precedes conditioned freezing (Kinsky et al. 2025), indicating that these HPC processes may be essential for memory consolidation. Together, these findings demonstrated that protein synthesis in the HPC is critical during the early stages of memory consolidation and may drive the functional reorganization of HPC circuits, though they did not establish a necessary role for synaptic plasticity in long‐term memory storage.

LTP in the HPC has long been associated with learning and memory, but direct evidence for its necessity in memory encoding and recall was limited due to a lack of appropriate tools. A recent study addressed this gap using eGRASP, a viral toolkit that enables fluorescent detection of synaptic connections between genetically defined pre‐ and postsynaptic cells. Two days after CFC, the study found selectively enhanced synaptic connectivity between CA3 and CA1 engram cells (Choi et al. 2018) (Figure 1C). This enhancement involved the formation of new spines on sparsely innervated regions of CA1 engram cell dendrites and increased spine clustering (Lee, Lee, et al. 2023) (Figure 1C)—a phenomenon previously linked to successful learning and memory (Frank et al. 2018). The extent of plasticity was correlated with memory strength, and LTP was occluded in engram cells, suggesting that hippocampal LTP is critical for memory.

Another study directly tested the causal role of LTP using a technique termed chromophore‐assisted light inactivation (CALI) (Goto et al. 2021). CALI blocks the accumulation of cofilin in dendritic spines, thereby preventing spine enlargement, a structural change that occurs during LTP (Goto et al. 2021). Using CALI to block structural LTP within 20 min after learning an inhibitory avoidance task impaired memory recall the following day (Figure 1A). This manipulation also prevented the emergence of selective encoding of the conditioned chamber, demonstrating that ‘online’ LTP that occurs during and immediately after learning is crucial to establish a recent memory (Goto et al. 2021). Together, these studies showed that engram cells exhibit preferential connectivity within days of learning and provided compelling evidence that hippocampal LTP is required for long‐term memory formation.

After associative learning, sleep‐dependent processes further promote memory consolidation. Long‐range oscillatory activity and HPC replay during sleep support memory consolidation (Atherton et al. 2015; Chen and Wilson 2017; Girardeau et al. 2009; Wilson and McNaughton 1994), but the specific molecular programs, cells, and circuits involved are only beginning to be understood. Using CALI to disrupt ‘offline’ LTP occurring during sleep—starting 2 h after learning—impaired memory recall the following day (Goto et al. 2021). Unlike the effects of blocking online LTP, which occurred during learning, blocking ‘offline’ LTP prevented the formation of synchronously active cell assemblies. CALI had no effect when administered 24 h after learning, suggesting HPC ‘offline’ LTP only occurs during the first day after associative learning.

Another study found that post‐learning sleep deprivation led to subregion‐specific changes in HPC engram cell activity and gene expression (Wang et al. 2024). Sleep deprivation increased reactivation of engram neurons in the CA fields and in the superior blade and hilus of the DG, but reduced reactivation in the inferior blade. This reduction was linked to suppressed neuronal activity and disrupted learning‐induced gene expression, particularly in genes associated with cytoskeletal remodeling (Wang et al. 2024) (Figure 1B). Additionally, consolidation of HPC‐dependent memories exhibits diurnal fluctuations, with mice showing better long‐term memory performance during the day than at night (Bellfy et al. 2023). These differences were associated with oscillating levels of the circadian clock gene *Period1 (Per1)*, suggesting that circadian genes play unexpected roles in regulating HPC memory consolidation (Bellfy et al. 2023). Together, these studies revealed novel hippocampal mechanisms through which post‐learning sleep influenced memory consolidation.

More recent work has challenged aspects of traditional Hebbian models, revealing that memory engrams are highly dynamic. A study using electron microscopy to reconstruct synaptic connections between CA3 and CA1 engram cells 1 week after fear conditioning found that, contrary to Hebbian predictions, engram cells were primarily connected to non‐engram cells (Uytiepo et al. 2025). Although the total number of synapses did not change, CA3 engram cells expanded their network by increasing the number of multi‐synaptic boutons that contacted more than one CA1 cell (Uytiepo et al. 2025). Consistent with this, another study showed that engram composition in the DG begins to change within hours after learning, with neurons being systematically added to and removed from the engram (Tomé et al. 2024) (Figure 1B,C). Using a combination of computational and experimental approaches, the authors demonstrated that excitatory synaptic plasticity alone can drive dynamic engram formation, but inhibitory synaptic plasticity is necessary for engram selectivity (Tomé et al. 2024). Together with the work described above, these studies suggest that while Hebbian plasticity between engram cells may operate in the hours and days after learning, HPC engrams are more dynamic than previously thought. Future studies can examine how the expanded synaptic networks observed a week after learning contribute to long‐term memory storage and retrieval.

While the studies discussed thus far focused on intra‐HPC mechanisms that were important for memory consolidation, emerging research is beginning to illuminate how long‐range HPC–cortical circuits contribute to memory process. One recent study showed that G‐protein signaling pathways in astrocytes differentially affected memory recall (Figure 1A,D). Activation of the *G*_*q*_ signaling pathway in CA1 astrocytes during learning enhanced recent memory and promoted recruitment of CA1–mPFC projection neurons into the memory engram (Refaeli et al. 2024). In contrast, activation of *G*_*i*_ signaling during learning selectively impaired remote memory by disrupting CA3–CA1 communication, thereby reducing functional recruitment of the CA1–mPFC pathway (Refaeli et al. 2024). These astrocytic signaling pathways can be triggered by different neurotransmitters (e.g., *G*_*q*_ by acetylcholine and *G*_*i*_ by gamma aminobutyric acid (GABA)), and increase intracellular Ca^2+^ with different timescales (*G*_*q*_ activation leads to prolonged Ca^2+^ elevations, whereas the effects of G_i_ signaling subside more rapidly) (Chai et al. 2017; Kol et al. 2020). These differences may underlie the distinct contributions of *G*_*i*_ and *G*_*q*_ pathways to recent and remote memory.

The subiculum contains RSC‐projecting neurons that uniquely express the vesicular glutamate transporters VGLUT1 or VGLUT2 and mediate recent contextual memory retrieval and long‐lasting memory storage, respectively (Yamawaki, Corcoran, et al. 2019) (Figure 1A). While most studies have focused on a single level of analysis—molecular, synaptic, cellular, or systems—future work integrating across these levels will be essential to uncover how and when synaptic plasticity in long‐range circuits mediates the transfer of memories from the HPC to the neocortex.

### 2.2 Retrosplenial Cortex

The RSC plays a key role in spatial and contextual memory (Vann et al. 2009; Miller et al. 2014) and is thought to promote systems consolidation by facilitating the transfer of information from the HPC to the PFC (Vann et al. 2009). The RSC comprises three major subregions: the granular regions *a* and *b* (RSCga, RSCgb; Area 29) and the dysgranular region (RSCdg, Area 30) (Vann et al. 2009; van Groen and Wyss 1990, 1992, 2003). Although RSCga, RSCgb, and RSCdg are highly interconnected, they exhibit distinct anatomical and functional properties, including differences in the cytoarchitecture of pyramidal cells across layers (Vogt and Paxinos 2014). The RSCg resembles the three‐layered archicortex of the HPC, whereas the RSCdg more closely resembles the six‐layered neocortex (Miller et al. 2014). Consistent with these cytoarchitectonic distinctions, RSCg is considered part of the limbic system due to its extensive connectivity with the ATN and HPC, while Rdg is more neocortical, with preferential connections to the visual cortex, parietal cortex, and the parahippocampal region (Miller et al. 2014). Polysynaptic pathways linking the visual cortices, RSC, and the HPC may initially convey sensory information that is integrated into contextual representations, whereas reciprocal RSC–HPC connections may contribute to subsequent memory consolidation and stabilization (Miller et al. 2014).

Due to its specific connectivity, the RSC is essential for spatial navigation, spatial memory, and contextual memory (Vann et al. 2009; Miller et al. 2014). Here, we focus on its role in long‐term memory. Substantial evidence shows that RSC is involved in the expression of both recent and remote contextual fear memories (Figure 1C,D). In rodents, electrolytic and neurotoxic lesions in RSC at the time of learning impaired contextual fear memories without affecting acquisition or cued fear (Keene and Bucci 2008a, 2008b). Reversible inactivation of RSC disrupted both recent and remote memory retrieval. The RSC also exhibited increased expression of IEGs following context exposure, CFC (Baumgärtel et al. 2018; Robinson et al. 2012), and especially after remote memory retrieval (Maviel et al. 2004). Optogenetic inhibition of anterior RSC during trace fear conditioning*—*in which there is a temporal separation between the cue and the shock*—*disrupted later freezing to a conditioned cue, whereas inhibiting posterior RSC impaired later contextual freezing (Trask, Pullins, et al. 2021). RSC lesions also impaired acquisition of active avoidance in a shuttle box assay (Lukoyanov and Lukoyanova 2006). Collectively, these findings suggested that the RSC is required for learning spatial relationships between threat and safety in contexts where behavioral strategies can be implemented to avoid danger, but not for acquisition of stimulus‐shock associations. Instead, RSC activity during CFC is required to establish a lasting memory of those learned associations (Figure 1A).

Recent studies have begun to reveal the cellular and molecular processes initiated in RSC during learning that are critical for establishing a lasting memory. Protein synthesis in the RSC during learning is required for subsequent memory retrieval (Figure 1A). For example, studies showed that protein synthesis in the RSC during inhibitory avoidance training was necessary for memory retrieval at both 2 and 7 days post‐training (Katche, Dorman, Slipczuk, et al. 2013). Blocking protein synthesis during trace fear conditioning also impaired recent memory retrieval of both context and cues (Kwapis et al. 2015). Additionally, a late phase of protein synthesis, occurring approximately 12 h after learning, is required for a corresponding increase in Fos expression in both RSC and in the HPC. Fos expression in the RSC was, in turn, necessary for the formation of long‐lasting memories (Katche, Dorman, Gonzalez, et al. 2013) (Figure 1B). Consistent with this, overexpression of CREB–an upstream regulator of Fos–in the RSC during learning enhanced subsequent spatial memory performance (Czajkowski et al. 2014) (Figure 1A). Fos‐expressing RSC neurons activated during spatial or contextual memory acquisition were reliably reactivated during memory retrieval (Czajkowski et al. 2014; Cowansage et al. 2014; Tayler et al. 2013) and were sufficient to drive fear memory retrieval (Cowansage et al. 2014; Sehgal et al. 2025) (Figure 1A). Moreover, activation of those neurons during sleep accelerated systems consolidation, promoting a switch from HPC‐dependent to cortex‐dependent memory retrieval (de Sousa et al. 2019) (Figure 1B). Taken together with functional inactivation experiments, these studies suggest that protein synthesis and IEG expression in the RSC during and shortly after learning are critical for memory consolidation, but not for memory acquisition.

IEGs like Fos and Zif268 regulate the expression of synaptic proteins and drive synaptic plasticity (Minatohara et al. 2016). In line with this, fast excitatory synaptic transmission, synaptic plasticity, and neuromodulation in the RSC are critical for memory consolidation and retrieval. GluN2A subunit‐containing N‐methyl‐D‐aspartate receptor (NMDAR) activity was critical for both recent and remote contextual memory retrieval, but not for memory acquisition or cued fear (Corcoran et al. 2011) (Figure 1C). RSC α‐amino‐3‐hydroxy‐5‐methyl‐4‐isoxazolepropionic acid receptor (AMPAR) activity was required to retrieve a spatial memory (Czajkowski et al. 2014). Dendritic spine turnover and clustering were important for stabilizing lasting memories. In the RSC, dendritic ‘hotspots’ with naturally elevated levels of spine turnover facilitated the formation of learning and memory‐related spine clusters. Clustering occurred through an NMDA‐dependent mechanism and served to stabilize contextual fear learning‐related structural plasticity (Figure 1D). More clustering was associated with stronger associative learning. Modeling revealed that having more dendritic segments with high levels of spine turnover was associated with increased network sparsity, which may support discrimination and increase memory capacity (Frank et al. 2018). Dopamine (DA) receptor signaling in RSCa was both necessary and sufficient for long‐term memory maintenance (Katche, Dorman, Gonzalez, et al. 2013; de Landeta et al. 2022), and DAergic inputs from the ventral tegmental area to the anterior RSC may play an important role in memory consolidation (de Landeta et al. 2022) (Figure 1A). In other cortical areas, DA modulates neuronal activity and plasticity via interactions with NMDA receptors (Gee et al. 2012; Robinson and Sohal 2017). Thus, DA and NMDA signaling may jointly regulate learning‐related synaptic plasticity in the RSC that contributed to the formation of lasting memory engrams, enabling the brain to reliably link salient cues with appropriate behavioral responses.

Future studies can use intersectional viral‐genetic approaches to investigate the relationships between RSC activity and connectivity underlying memory processing. Recent work revealed that within the RSC, anatomically and genetically defined neurons participated in distinct subcircuits. For instance, RSC neurons that project to secondary motor cortex (M2) received greater input from the dorsal subiculum, thalamus, and sensory cortex, whereas AD‐projecting RSC neurons received greater input from local RSC neurons and the medial septum (Lin et al. 2024). Also, glutamatergic RSC neurons received preferential input from the isocortex, hypothalamus, midbrain, and hindbrain (Li et al. 2025). In contrast, GABAergic neurons received little input from those regions and instead received most of their synaptic input from the cortex, including from other RSC neurons (Li et al. 2025). Determining which RSC subcircuits are active during memory acquisition, consolidation, and retrieval will allow researchers to better understand how memory persistence and specificity change across the lifespan, as we discuss later in the review.

### 2.3 Prefrontal Cortex

The mPFC is essential for long‐term memory (Frankland and Bontempi 2005, 2006; Maviel et al. 2004; Frankland et al. 2004; Takashima et al. 2006) and plays key roles in both memory consolidation and retrieval. Physical and pharmacological lesions as well as optogenetic manipulations have demonstrated that mPFC activity is required for remote memory retrieval and for recent memory retrieval in some situations (Corcoran and Quirk 2007; Do‐Monte et al. 2015). The mPFC receives direct synaptic input from sites of memory encoding, including the HPC and the basolateral amygdala (BLA) (DeNardo et al. 2015; Hoover and Vertes 2007). Through these connections, the mPFC integrates information about learned associations and influences aspects of memory retrieval through its many long‐range projections. As we discuss below, it is also becoming increasingly clear that functional tagging of mPFC neurons at the time of learning is critical for memory consolidation.

Dynamic processes initiated in mPFC neurons during learning are important for consolidating a long‐term memory trace. There is substantial evidence supporting a model in which mPFC engram neurons are tagged during learning and then mature to support memory consolidation and remote memory retrieval (Lesburguères et al. 2011; Redondo and Morris 2011). During the weeks after memory encoding, mPFC engram neurons grow new dendritic spines, are more likely to be reactivated by conditioned stimuli, and their activity is required for memory‐induced freezing behavior (Figure 1D). This process depends on input from the medial EC during learning, suggesting that activity in this pathway during learning initiates a process of maturation. Offline structural LTP in mPFC that occurs 2 days after fear conditioning, but not 1 or 25 days after, was necessary for subsequent memory retrieval (Goto et al. 2021) (Figure 1B). In line with this, memory consolidation was associated with selective strengthening of the synaptic connections between mPFC engram cells that were active during learning (Lee, Kim, et al. 2023). This plasticity was CREB‐dependent and required sustained hippocampal input that could be conveyed by RSC (Lee, Kim, et al. 2023) (Figure 1B). Noradrenergic projections from the locus coeruleus (LC) acted through β1‐adrenergic receptors to regulate mPFC engram cell tagging during learning and subsequent memory consolidation (Fan et al. 2022) (Figure 1A). Together these studies indicated that synaptic strengthening of engram cells underlies memory consolidation and is driven by long‐range inputs from the HPC, cortex, and neuromodulatory centers.

Similar to what has been observed in the HPC, there is increasing evidence that mPFC engrams are dynamic, and new neurons and circuits are recruited to a memory trace over time. For instance, distinct long‐range projections are involved in memory retrieval at recent and remote timepoints. mPFC projections to the BLA were critical for recent but not remote cued fear, whereas mPFC projections to the paraventricular thalamus were required to retrieve remote but not recent cued fear memories (Do‐Monte et al. 2015). On the other hand, mPFC‐BLA projections were required for remote but not recent contextual fear memory retrieval Kitamura et al. (2017). As distinct classes of mPFC neurons project to different targets (Anastasiades and Carter 2021; Gao et al. 2022; Gongwer et al. 2023), these findings suggested that a functional reorganization of mPFC microcircuits is critical to establish remote memories. In line with this, new mPFC neurons are recruited to a memory engram across weeks. These cells were necessary for remote memory retrieval and their recruitment depended on mPFC activity at the time of learning (DeNardo et al. 2019). Compared to those active during recent memory, mPFC neurons that were active during remote memory retrieval had enhanced functional connectivity with cortical association areas (DeNardo et al. 2019). Together, these studies indicated that memory consolidation involves dynamic changes in the cellular composition of the mPFC engram, and may also involve target‐specific strengthening of mPFC afferent pathways (Figure 1D).

Recent studies have begun to uncover the mPFC molecular pathways that are important for memory consolidation. One recent study examined differentially expressed genes in mPFC neurons that were active during remote fear memory retrieval. While a range of neuronal cell types were activated, the differentially expressed genes were associated with vesicular exocytosis, transmembrane transport, dendritic spine organization, and long‐range intracellular transport (Chen et al. 2020). Enhanced membrane fusion and vesicle exocytosis may therefore be a critical mode of synaptic strengthening during memory consolidation. Glial cells also exhibited specific patterns of gene expression associated with remote memory retrieval, consistent with emerging evidence that glia support long‐term memory (Sun et al. 2024; Williamson et al. 2025). In astrocytes, upregulated genes were enriched in metabolic functions, suggesting that enhanced metabolic support from astrocytes may play a key role in memory consolidation (Figure 1D). This study also identified upregulated genes encoding partner synaptic adhesion molecules in neurons and astrocytes suggesting that astrocytes may serve to maintain synaptic strength to support memory consolidation (Chen et al. 2020).

### 2.4 Anterior Thalamic Nuclei

The ATN are a cluster of nuclei that serve as key nodes in memory consolidation circuits. They have reciprocal connectivity with the HPC, RSC, and mPFC (O‘Mara 2013). ATN activity is necessary for fear memory encoding (Yamawaki, Li, et al. 2019), consolidation (Toader et al. 2023), and remote memory retrieval (Vetere et al. 2021). Within the ATN, the anterodorsal (AD) nucleus primarily projects to the pre‐subiculum (PreSub) and RSC (Yamawaki, Li, et al. 2019). The anteroventral (AV) thalamus projects to the RSC but not the PreSub (Roy et al. 2021). Although both subregions receive input from similar brain areas, the AV receives unique input from the prelimbic cortex. Notably, while most regions send more input to the AV than to the AD, the RSC preferentially projects to the AD (Roy et al. 2021). These distinct connectivity patterns likely underlie subregion‐specific functions. The AD is critical for contextual memory encoding, whereas the AV regulates memory specificity (Roy et al. 2021). New studies described in this section have revealed specific roles for these subregions and their long‐range connections in memory consolidation.

Bidirectional interactions between the ATN, HPC, and RSC are vital for long‐term memory. Inhibition of AD during memory encoding, but not immediately afterward, disrupted performance in an inhibitory avoidance assay. Learning increased excitatory synapse numbers, Fos expression, and both theta and gamma oscillatory power in the AD (Figure 1A). Recent work revealed that these learning‐related changes are circuit specific. The AD‐RSC circuit, but not the AD‐PreSub circuit, exhibited post‐encoding synaptic plasticity and enhancement in vivo local field potential (LFP) coherence. AD activity also drove a learning‐related enhancement of Fos in both CA1 and in RSC, particularly in RSC neurons that project to the EC. Activity in this AD‐RSC‐EC disynaptic circuit was required during encoding for successful memory recall (Figure 1A).

In contrast, the AV‐RSC circuit regulates memory specificity, but is not necessary for memory recall. AV connections to vasoactive intestinal peptide (VIP) + interneurons in RSC mediated these effects. Hippocampal and ATN inputs converged on the distal dendritic tufts of granular layer 5 RSC neurons to regulate long‐term memory (Yamawaki, Li, et al. 2019). The HPC neurons involved in this circuit were GABAergic, arising from the stratum radiatum‐lacunosum molecular border in dorsal CA1 (Jinno 2009; Rock et al. 2018). These inhibitory projections from CA1 and excitatory projections from ATN innervated the distal tufts of the same RSCg neurons and played opposing roles in memory. Inhibiting the GABAergic CA1‐RSC circuit during encoding enhanced contextual memory performance, while inhibiting the ATN‐RSC impaired it. These findings suggest that the two pathways work in opposition to dynamically regulate the memory‐encoding functions of the RSC. Collectively, these studies illustrated how distinct ATN‐RSC subcircuits control different aspects of long‐term memory.

ATN‐mPFC interactions are also crucial for memory consolidation. Over time, consolidation is accompanied by a gradual increase in context‐specific correlations between ATN and mPFC activity. The ATN preferentially encodes salient experiences, and increasing gain in the ATN to anterior cingulate cortex pathway enhances memory consolidation. This increased ATN‐mPFC functional coupling coincided with stronger intra‐mPFC ensemble correlations during the retrieval of salient memories. These findings suggest that ATN selects salient memories and reorganizes them into the cortex. Similar to observations in the RSC, activity in the ATN‐mPFC pathway during learning was critical for establishing coordinated ensemble activity in ACC and for the behavioral expression of a remote memory (Toader et al. 2023). Future studies can leverage engram cell tagging and manipulation strategies to further explore when and how activity‐dependent programs are engaged in the ATN, and how these programs relate to other brain regions.

## 3 The Maturation of Memory Systems

Compared to memories formed in adulthood, those formed early in life are often more generalized and rapidly forgotten (Ramsaran et al. 2018, 2023). These phenomena—known as infant generalization (IG) and IA, respectively—are conserved across species, suggesting they represent fundamental aspects of brain development (Ramsaran et al. 2018). Investigating the mechanisms underlying the maturation of memory systems is critical, as early life experiences are known to shape neuronal development and behavior across the lifespan. For example, environmental enrichment can accelerate eye opening and enhance contextual memory recall in juveniles (Xue et al. 2024). Conversely, adverse rearing conditions can hasten hippocampal development, leading to the early emergence of persistent memory (Callaghan and Richardson 2011; Cowan et al. 2013). Individuals exposed to early adversity are at greater risk for developing fear and anxiety disorders, raising the possibility that IA may protect against developing behavioral disorders.

Memory specificity develops alongside memory persistence during early life. Striking the right balance between memory specificity and generalization is essential for guiding appropriate behavior across different contexts. While memory generalization can support adaptive behaviors in novel or changing environments, excessive or inappropriate generalization—such as that observed in individuals with post‐traumatic stress disorder—can be maladaptive. For developing animals, exposure to novel contexts is particularly significant, as most early life experiences occur without prior memories to inform behavior. During development, IG might serve as an adaptive feature of early memory development, enabling young animals to navigate and learn from unfamiliar environments (Ramsaran et al. 2018). To better understand sensitive periods for memory system maturation—and how interventions might protect at‐risk individuals—it is essential to study how memory systems mature in the typically developing brain. Below, we highlight recent work that begins to uncover the circuit mechanisms underlying IG and IA.

### 3.1 Hippocampus

**FIGURE 2:**
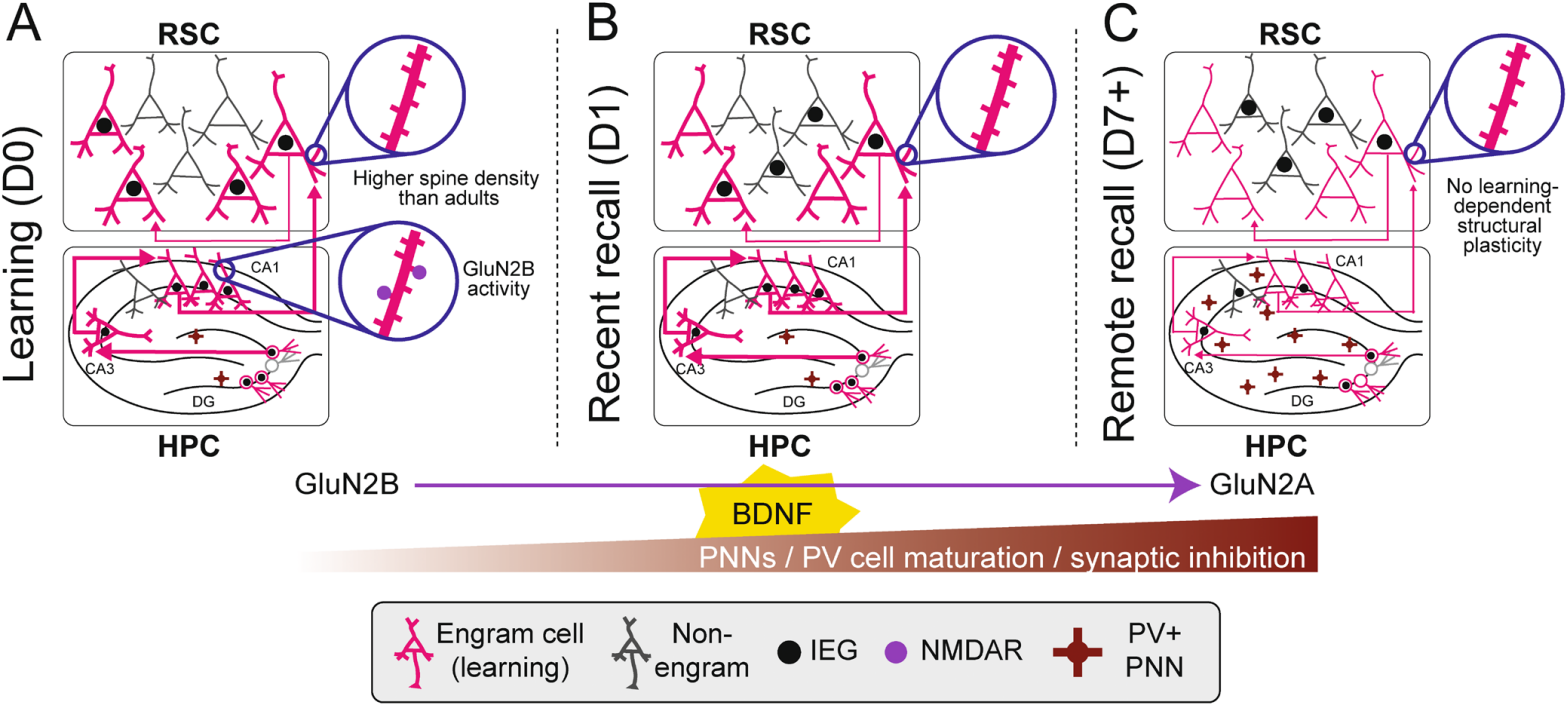
The roles of engram cells in memory encoding, consolidation, and retrieval in development. Summary of recent findings on engram cells in the HPC and RSC based on experiments conducted in ~P17–P20 rodents. (A) During learning, RSC neurons have high spine density compared to adults, engrams are dense in the RSC and HPC, and GluN2B activity is required for learning. (B) During recent memory retrieval, RSC neurons still have high spine density compared to adults and HPC engrams are dense. (C) During remote memory recall, RSC neurons still have higher spine density than adults, RSC engrams are unstable (infrequent reactivation of original engram cells), and HPC and RSC engrams are silent.

Most studies on memory system development have focused on HPC engrams. There is strong evidence that hippocampal neurogenesis plays a significant role in IA. The continuous addition of new neurons to the hippocampal circuits creates competition with existing neurons, alters synaptic weights, and refines circuits—processes that can ultimately disrupt previously stored memories. Although neurogenesis occurs throughout life, the rate is significantly elevated during infancy. In mice, higher rates of neurogenesis were negatively correlated with memory persistence, and experimentally increasing hippocampal neurogenesis in adult mice disrupted memory recall (Akers et al. 2014). Conversely, reducing neurogenesis in infant mice enhanced memory persistence (Figure 2), demonstrating that high levels of neurogenesis in the developing brain contribute to IA.

To understand the neural basis of IA, it was first necessary to determine whether infant memories are erased or instead encoded but rendered inaccessible. Recent studies support the latter, showing that memories are indeed encoded in the infant HPC and can be reinstated through strong reminder shocks or optogenetic reactivation of HPC engram cells (Guskjolen et al. 2018; Power et al. 2023; Travaglia et al. 2016). Moreover, infant engrams can be updated, much like adult memory traces (Zaki et al. 2025), leading to the permanent reinstatement of a once‐forgotten memory (Power et al. 2023). These findings indicate that memories formed in infancy are stored in the HPC but quickly become inaccessible. Emerging evidence from human infants supports a similar model. The HPC begins to encode memories around 1 year of age (Yates et al. 2025), suggesting that IA reflects developmental differences in memory consolidation or post‐encoding processes.

While the formation of a HPC engram is critical for subsequent memory consolidation, retrieval, and specificity, the process of engram allocation during development—and how it relates to memory specificity—is only beginning to be understood. In adults, neuronal excitability determines whether cells are allocated to an engram (Mocle et al. 2024), and sparse engrams are essential for mediating memory specificity (Ramsaran et al. 2018, 2023). Recent work in the CA1 region of the HPC revealed that juvenile engrams were more densely populated than adult engrams (Ramsaran et al. 2023). Reducing neuronal excitability during juvenile learning resulted in sparser HPC engrams and enhanced memory specificity, suggesting that broader engrams—driven by elevated HPC activity—contribute to IG. The same study also implicated immature inhibitory circuits. In juvenile mice, immature parvalbumin (PV) + interneuron networks allowed broader engram formation during infant learning, contributing to IG (Ramsaran et al. 2023) (Figure 2A).

The maturation of PV+ interneurons depends on the development of perineuronal nets (PNN), components of the extracellular matrix that stabilize connections between excitatory and inhibitory cells in both the HPC and cortex. By the fourth postnatal week in mice, PV+ interneurons and PNN reached adult‐like levels, which served to restrict the size of the engram, enhancing memory specificity (Ramsaran et al. 2023) (Figure 2A). While PNNs have previously been shown to contribute to circuit development in sensory cortical areas, these findings suggest their role extends to cognitive and emotional systems, where they regulate engram cell allocation and contribute to the transition from generalized to specific memory.

Infancy has been proposed as a sensitive period in HPC development during which experiences can shape the maturation of memory systems (Travaglia et al. 2016). New studies are revealing the cellular and molecular mechanisms through which environmental factors influence developing HPC circuits. While it has long been recognized that PNN development in the cortex is driven by sensory experience, thereby guiding cortical maturation (McRae et al. 2007), recent research shows that HPC PNNs also contribute to experience‐dependent development (Ramsaran et al. 2024). Early life deprivation in the form of maternal separation and early weaning delayed the formation of PNNs in CA1, whereas early life enrichment accelerated CA1 PNN development and enhanced memory specificity. Importantly, administration of brain‐derived neurotrophic factor (BDNF) rescued impaired PNN development following ELA and restored age‐appropriate memory specificity. These findings reveal how early life experiences can regulate the timing for cognitive development by modulating PNNs.

BDNF plays multiple roles in HPC development and also influences IA. It promotes an experience‐dependent developmental switch in NMDAR subunit expression, favoring GluN2A over GluN2B (Travaglia et al. 2016). These subunits contribute differentially to NMDAR current kinetics and synaptic plasticity (Liu et al. 2004) and memory formation in infant rats requires GluN2B but not GluN2A (Travaglia et al. 2016) (Figure 2A). BDNF administration rescues memory impairments associated with IA (Travaglia et al. 2016; Ramsaran et al. 2024). Together, these data suggest that the HPC has a sensitive period during which BDNF regulates both NMDAR subunit composition and PNN development, thereby guiding synaptic maturation and the experience‐dependent development of memory functions.

In a mouse model of maternal immune activation (MIA)–a distinct form of altered early experience–HPC engram size and spine plasticity were enhanced, and infant mice did not exhibit IA (Power et al. 2023). MIA is associated with increased risk for developing autism spectrum disorder, which is often accompanied by memory deficits (Liu et al. 2023). However, a study recently reported that autistic individuals have exceptionally vivid memories from early in life (Zamoscik et al. 2016). Still, the effects of MIA on IA in humans are poorly understood and require further study. Future studies should investigate whether BDNF‐dependent mechanisms contribute to these effects and explore whether modulating these pathways could offer therapeutic potential for individuals at risk for neurological and behavioral disorders due to altered hippocampal development.

### 3.2 Retrosplenial Cortex

The RSC is known to play an important role in memory formation and consolidation in the adult brain, but its developmental trajectory remains poorly understood. The parahippocampal region (PHR) has extensive connections with the RSC, and bidirectional communication between the RSC and hippocampal formation is thought to be critical for memory encoding and consolidation. In rats, the first fibers from RSC to PHR arrive at birth (Sugar and Witter 2016), and topographically organized projections to the parasubiculum, PreSub, and EC reach adult‐like densities by P12 (Sugar and Witter 2016).

While the standard systems consolidation hypothesis proposes that remote memory recall in adulthood increasingly relies on cortical regions rather than the HPC, little is known about how systems consolidation unfolds during development—or whether differences in this process contribute to IA. Connectivity among regions implicated in memory consolidation continues to mature into early adulthood (Sugar and Witter 2016; Haugland et al. 2019; Klune et al. 2021), and this delayed maturation could contribute to IA. A recent brain‐wide Fos‐based screen found that functional maturation of the neocortex coincides with the transition from IA to persistent memory (Jin et al. 2024). In this study, several interconnected cortical regions known to support memory consolidation in adults—including the RSC, temporal association area, and ectorhinal cortex—exhibited less learning‐dependent activation in infants. Instead, infants showed increased learning‐related activation in olfactory, HPC, and hypothalamic regions (Jin et al. 2024).

The same study showed that the RSC encodes infant memories and that activating RSC engram cells can restore forgotten memories. However, RSC engrams were larger but less stable compared to adults. This study suggests that infant memories are also encoded in the neocortex, in larger engrams as in the HPC, and that immature neocortical synaptic connectivity may reduce the likelihood that engram cells are naturally reactivated, contributing to IA (Jin et al. 2024) (Figure 2B,C). Future studies should investigate the role of inhibitory circuitry and PNNs in the development of RSC engrams and their relationship to IA.

Dendritic spine growth is a key feature of long‐term plasticity and has been linked to learning and memory processes in the RSC (Frank et al. 2018; Baumgärtel et al. 2018). In adults, spine turnover is relatively balanced, with approximately 5% of spines gained and lost over time (Frank et al. 2018). Recent studies in infant mice revealed that RSC engram cells in fear conditioned mice exhibited similar spine densities to those of non‐shocked control mice, and both groups had higher spine densities than adult RSC engram cells (Jin et al. 2024). This suggests that early in development, hyperconnectivity may contribute to noisy encoding and unreliable engram reactivation. Rapid spine dynamics—on the scale of minutes to hours—have not yet been studied in the developing RSC. However, in other developing cortical areas, such as the barrel cortex and mPFC, younger mice lose more spines (~20%) than they gain (~5%–10%, depending on the region), which may destabilize nascent neural networks (Boivin et al. 2018; Zuo et al. 2005). While these findings suggest that early in life, dense—and perhaps non‐specific—connectivity of RSC engram cells contributes to IA, future studies are needed to determine whether the RSC engram neurons in infants also have highly dynamic spines that contribute to forgetting.

Understanding how the maturation of neocortical regions like the RSC contribute to the development of memory is essential in determining why infant memories become inaccessible over time. It remains unclear whether and how adult‐like consolidation mechanisms influence RSC engrams during development. Investigating how RSC engrams form and communicate via long‐range projections to the mPFC and other cortical regions will be critical to determining to what extent systems consolidation occurs in the infant brain—and if so, how it differs from that in adults.

### 3.3 Prefrontal Cortex

While the mPFC has a well established role in long‐term memory in adults, relatively few studies have examined its contributions to the maturation of memory functions. However, understanding developmental trajectories in mPFC circuits can provide important insight into how network integration evolves during infancy. In the first few postnatal days in rodents, the mPFC undergoes rapid pyramidal cell growth, axon lengthening, and increasing spine density (Kroon et al. 2019). During this period, younger rodents show increased spontaneous neural activity (Brockmann et al. 2011), increased IEG expression (Jia et al. 2018), and layer‐specific shifts in the balance of excitatory and inhibitory signaling (Kroon et al. 2019). Long‐range projections from the mPFC to memory‐relevant regions—such as the BLA and EC—are established around the end of the first postnatal week and continue to mature through adolescence (Arruda‐Carvalho et al. 2017; Hartung et al. 2016; Klune et al. 2025). Identifying molecular mechanisms underlying mPFC development will be critical to understanding the delayed maturation of these regions in the context of memory system development.

Recent studies have begun to address the mPFC's role in memory during early life. In infant rodents, Fos expression increased in the HPC following CFC, but not in the mPFC (Fan et al. 2022). Similarly, compared to adolescents and adults, juvenile mice showed reduced mPFC activity during threat‐conditioned stimuli, and mPFC‐amygdala circuits did not acquire their adult‐like roles in fear‐guided threat avoidance until after adolescence (Klune et al. 2025). Together, these findings suggest a more passive role for the mPFC in learning during infancy and early juvenile stages.

One recent study suggested that infantile spatial experiences can influence memory formation later in life (Contreras et al. 2024). Infant rats exposed to spatial exploration—experiences that are later forgotten—demonstrated improved performance on object‐place recognition tasks in adulthood. According to systems consolidation theory, retrieval shortly after encoding (e.g., 3 h later) is primarily HPC‐dependent, with minimal involvement of the mPFC (Frankland and Bontempi 2005). Surprisingly, adult rats with early spatial experience showed greater Fos expression in the mPFC than in the HPC during retrieval 3 h later. Inhibiting mPFC activity in adulthood eliminated the memory enhancement conferred by early life spatial experience. Notably, sleep deprivation in infancy was required for this enhancement to emerge, suggesting a complex interaction between early experience, sleep, and mPFC maturation. These findings position the mPFC as a promising target for future studies on IA and the developmental regulation of memory.

## 4 Conclusion and Future Directions

Here we summarized recent studies examining how engram cells in four key brain regions contribute to memory encoding, consolidation, retrieval, and forgetting across development and into adulthood. In adults, HPC engrams are more dynamic than previously thought. In the week following learning, engram cells form new synaptic connections with non‐engram cells (Uytiepo et al. 2025), and additional neurons are incorporated into the HPC engram through excitatory synaptic plasticity (Tomé et al. 2024). In the RSC, offline processes involving protein synthesis (Katche, Dorman, Slipczuk, et al. 2013; Kwapis et al. 2015), IEG expression (Katche, Dorman, Gonzalez, et al. 2013; Czajkowski et al. 2014), and dopamine signaling (de Landeta et al. 2022) mediate memory consolidation. Over the subsequent weeks, engram cells in the mPFC functionally mature (Lesburguères et al. 2011; Redondo and Morris 2011), additional mPFC neurons are recruited to the memory engram (Do‐Monte et al. 2015; Kitamura et al. 2017), and these neurons become essential for remote memory retrieval (DeNardo et al. 2019). Together, these findings suggest that memory consolidation relies on processes in HPC and RSC engram cells that occur within hours to days after learning, while critical changes in mPFC engram cells unfold over weeks. Recent studies also highlight the role of long‐range circuits connecting engrams in the HPC, RSC, mPFC, and ATN, which uniquely contribute to memory consolidation, retrieval, and specificity.

In the developing brain, infant memories are encoded—at minimum—within the HPC and RSC engram cells, and these silent engrams can persist into adulthood. There is strong evidence that increased hippocampal neurogenesis (Akers et al. 2014), immature synapses, and larger engrams with more allocated neurons (Ramsaran et al. 2023) contribute to IA and IG, respectively. However, due to the limited number of studies and the predominant focus on the HPC, the mechanisms underlying the retrieval failure of these silent engrams remain poorly understood. Here we discuss new findings of how RSC engram cells also encode latent infant memories (Jin et al. 2024) and suggest new hypotheses of how RSC circuits may contribute to IA through mechanisms that involve dense synaptic connectivity and non‐specific cell activation during memory formation. By integrating knowledge of adult memory consolidation with insights into memory circuit development, we can begin to predict how—and to what extent—the developing brain consolidates memories.

While differences in HPC memory encoding appear to contribute to IA, it is also critical to understand how memory consolidation mechanisms change across development. In adults, systems consolidation involves a transfer of information from the HPC to the neocortex (Tonegawa et al. 2018; Frankland and Bontempi 2005, 2006). This transition depends on local synaptic remodeling and long‐range interactions with the ATN and neocortex (Yamawaki, Li, et al. 2019). Although some of the key circuits involved in memory consolidation—such as those between the parahippocampal areas and the RSC—are established early in development (Sugar and Witter 2016), others, including long‐range ATN connections, remain unstudied in the developing brain. Future research should investigate the anatomical maturation of long‐term memory networks to better understand the structural basis for developmental changes in memory processing.

Recent work comparing brain‐wide IEG expression following CFC in infants, juveniles, and adults has provided new insights into how the functional organization of fear memory networks evolves with development. While the HPC remained a central network node across all ages, cortico‐cortical connections became more prominent in adults. Notably, functional connectivity between the subiculum and RSC exhibited a marked shift from negative to positive between infancy and the juvenile stage (Jin et al. 2025). These findings suggest that the protracted development of neocortical and hippocampal‐cortical connectivity may alter the mechanisms by which memories are consolidated across development. However, IEG expression varies depending on cell type and brain region (Arai et al. 2025; Chiaruttini et al. 2025), which can influence the apparent connectivity derived from IEG‐based maps. Therefore, future studies should also use anatomical circuit tracing and functional approaches (e.g., optogenetics) to investigate how the functional maturation of cortical association areas and their long‐range connections contributes to IA vs. memory persistence.

In adults, memory consolidation involves coordinated changes at the molecular, synaptic, cellular, and systems levels. For example, studies revealed that long‐range circuit activity together with molecular expression of CREB is critical for engram cell tagging, enhancements in cell‐type specific synaptic connectivity, and the functional maturation of engram cells (Lee, Kim, et al. 2023; Fan et al. 2022; Kitamura et al. 2017). IEG expression is required for synaptic plasticity and memory formation (Fleischmann et al. 2003; Hall et al. 2001; Jones et al. 2001). These processes occur online during learning and offline during subsequent periods of quiet wakefulness and sleep. However, their roles in the developing brain are not well understood. Although IEGs are expressed at higher levels in early life (Travaglia et al. 2016; Jia et al. 2018), how they regulate plasticity and memory in infants and juveniles is still an open question. Sleep‐dependent processes are known to play a key role in memory consolidation in adults (Goto et al. 2021; Atherton et al. 2015; Chen and Wilson 2017; Girardeau et al. 2009; Wilson and McNaughton 1994; de Sousa et al. 2019; Chang et al. 2025), yet sleep patterns during infancy and early childhood differ substantially from those in adulthood (Davis et al. 2004; Mason and Spencer 2022). How these differences impact memory consolidation during development has not been fully elucidated. Future studies using advanced viral‐genetic tools in the developing brain will be instrumental in uncovering how integrated molecular, cellular, and circuit‐level changes regulate memory consolidation across development.

Future studies stand to gain valuable insights by comparing the mechanisms of forgetting in aging and in Alzheimer's Disease (AD) with those underlying IA. Although these forms of forgetting occur at opposite ends of the lifespan, examining their similarities and differences may reveal core principles of forgetting, as well as age‐ and condition‐specific mechanisms. For instance, hippocampal engram studies in mouse models of AD suggest that memory retrieval deficits—rather than failures of encoding—play a central role in memory loss (Roy et al. 2016), paralleling current hypotheses about the basis of IA. In AD models, loss of dendritic spines is a potential contributor to retrieval failure. As synapse pruning is a critical aspect of development, loss of dendritic spines may be a common mechanism of amnesia across the lifespan. By directly comparing mechanisms of forgetting across infancy, adulthood, aging, and neurodegenerative conditions, we can better distinguish the fundamental processes that govern memory decline from those that arise through developmental or degenerative changes.

## Conflicts of Interest

The authors declare no conflicts of interest.

## Data Availability

We hereby confirm the absence of shared data associated with this manuscript.
